# NEIGHBORHOOD WALKABILITY IS ASSOCIATED WITH GPS-DERIVED LIFE-SPACE MOBILITY OF OLDER ADULTS

**DOI:** 10.1093/geroni/igac059.1011

**Published:** 2022-12-20

**Authors:** Kyle Moored, Breanna Crane, Pamela Dunlap, Michelle Carlson, Andrea Rosso

**Affiliations:** Johns Hopkins Bloomberg School of Public Health, Baltimore, Maryland, United States; Johns Hopkins Bloomberg School of Public Health, Baltimore, Maryland, United States; University of Pittsburgh, Pittsburgh, Pennsylvania, United States; Johns Hopkins Bloomberg School of Public Health, Baltimore, Maryland, United States; University of Pittsburgh, Pittsburgh, Pennsylvania, United States

## Abstract

For community-dwelling older adults, living in a more walkable neighborhood may encourage greater travel outdoors (i.e., life-space mobility). We assessed associations between neighborhood walkability and objective, GPS-derived life-space mobility. Participants were 149 adults (Age: M=77.1±6.5, 67% women) from a randomized trial to improve walking in older individuals. Participants carried a GPS at baseline that passively collected real-time location data for 5-7 days. Life-space mobility was quantified using median percentage time spent outside of home (pTOH). Neighborhood walkability was assessed using a modified Active Neighborhood Checklist and Google Street View, and a composite factor score was derived (M=-0.13±0.90, greater score=less slopes, more mixed residential/commercial land use). Each 1-point higher walkability score was associated with 2.5% greater pTOH (95%CI: 0.21-4.69, β=.23, p=.032), after adjusting for age, sex, race, device, season, and clustering on Census tract. Future work will examine how neighborhood walkability and functional status may interact to influence life-space mobility.

